# Endometrial Cut Off Thickness as Predictor of Endometrial Pathology in Perimenopausal Women with Abnormal Uterine Bleeding: A Cross-Sectional Study

**DOI:** 10.1155/2022/5073944

**Published:** 2022-01-04

**Authors:** Priti Kumari, Harsha S. Gaikwad, Banashree Nath

**Affiliations:** ^1^Department of Obstetrics and Gynaecology, VMMC and Safdarjung Hospital, Safdarjung Enclave, New Delhi 110029, India; ^2^Department of Obstetrics and Gynaecology, All India Institute of Medical Sciences, Raebareli, Uttar Pradesh, India

## Abstract

**Purpose:**

We aim to determine the predictive value of endometrial thickness by transvaginal ultrasonography (TVS) in diagnosing endometrial pathology and to evaluate whether Doppler complements its diagnostic efficacy in perimenopausal women with abnormal uterine bleeding.

**Methods:**

This cross-sectional observational study was conducted among 70 perimenopausal women with AUB who underwent TVS measurement of endometrial thickness (ET) and Doppler flow indices followed by endometrial sampling and histopathological examination (HPE).

**Results:**

In HPE, 51 (73%) women had normal diagnosis while 19 (27%) women had neoplastic histology either benign or malignant. They were categorised into group I and group II, respectively. There was a significant difference in age (*P*=0.001) and incidence of obesity (*P*=0.01) between the two groups. The ETs measured in group I and group II were 7.89 ± 2.62 mm and 14.07 ± 3.96 mm, respectively, with significant difference (*P* < 0.001). A TVS-ET of 10.5 mm had the highest sensitivity and specificity of 89.5% and 86.3%, respectively, PPV of 70.68%, NPV of 95.68%, LR+ of 6.52, and LR− of 0.12. Doppler flow velocimetric study of endometrial and uterine vessels did not demonstrate a significant difference.

**Conclusions:**

Women in perimenopause with AUB should be offered to undergo endometrial sampling for histopathological examination if TVS ET ≥10.5 mm. The coexisting risk factors especially higher age (>45 years) and obesity (BMI>30) significantly escalate the chances of developing endometrial pathology.

## 1. Introduction

The perimenopausal period or climacteric begins with the irregularity of the menstruation cycle and extends up to 1 year after permanent cessation of menses [1]. It refers to the time period in the late reproductive years generally in the late 40s to early 50s. During this climacteric period, menstrual cycles become occasionally anovulatory due to a gradual decrease in the recruitment of ovarian follicles with a subsequent decline in the level of oestradiol. This downturn of the hormonal milieu causes increased incidence of prolonged cycles of amenorrhoea alternating with heavy menstrual bleeding [2]. The presentation of abnormal uterine bleeding (AUB) in perimenopausal patients may include a spectrum of menstrual disorders [3].

Modalities for diagnosis have evolved from the traditional dilation and curettage (D and C) of the uterus to immunohistochemical markers, transvaginal ultrasound (TVS), colour Doppler, sonosalpingography, and hysteroscopy. Histopathology is still considered the gold standard technique. However, it is an invasive procedure with the risk of various complications. The discovery of a noninvasive or minimally invasive technique that is easily performed, accepted, and tolerated well by patients, cheap, highly sensitive, and specific to study the endometrial pathology is warranted. TVS is a reliable method with wide-ranging applicability and clinical efficiency whose acceptance by both healthcare providers and patients is widespread [4]. Evaluation of endometrial thickness by ultrasonic measurement has considerable significance in diagnosing benign and neoplastic endometrial lesions in women of all ages [5].

In women with postmenopausal bleeding, numerous studies have established TVS-ET as an initial screening procedure to ascertain whether a cut off limit for endometrial thickness can be proposed to rule out endometrial pathology [3, 6]. Endometrial stripe cut off values widely ranging from 3 to 14 mm have been suggested in various studies to detect endometrial pathology in premenopausal women also [7, 8]. There are, however, scarce studies undertaken to determine the cut off limit of ET in perimenopausal women for choosing patients to offer an invasive endometrial sampling. Along with TVS, the role of Doppler is debated with studies depicting conflicting results [9, 10].

Hence, we undertook this study and compared the findings of endometrial morphology by transvaginal sonography and colour Doppler in perimenopausal women with abnormal uterine bleeding and correlated the findings with the histopathological study of the endometrium, which remains the gold standard. We aim to determine the cut off limit for the endometrial thickness that will aid in ruling out endometrial pathology and evaluating whether Doppler complements the diagnostic efficacy of transvaginal sonographic evaluation of endometrium in perimenopausal women with abnormal uterine bleeding.

## 2. Materials and Methods

This cross-sectional observational study was conducted for a period of 18 months at a tertiary care centre in North India from October 2017 to April 2019 after obtaining approval from the Institutional Ethics Committee. Informed written consent was obtained from all participants.

### 2.1. Study Sample

Perimenopausal women in the age group above 40 years old with complaints of AUB within the study period and consenting to participate were recruited for the study. Women with ovarian tumour, tubo-ovarian mass, pregnancy, genital tract malignancy, AUB due to other causes like hormones or medications with the potential to affect pelvic blood flow, thyroid hormone disorders, coagulation disorder, and vascular malformation of the uterus were excluded from the study. All eligible cases were interviewed through a predesigned, semistructured questionnaire. They subsequently underwent complete general and gynaecological examination and preliminary transvaginal ultrasound.

### 2.2. Study Procedure

#### 2.2.1. Ultrasonographic Examination

TVS examinations were performed by one of the authors (H.S.) with a vaginal probe of 7–10 MHz of a Phillips HD11XE Ultrasound device to look for endometrial echo complex, uterine size, contour, and Doppler flow pattern of endometrial vessels in the same examination setting. Uterus and adnexa were scanned in longitudinal and transverse planes making note of any abnormality. The image of the uterus was longitudinally magnified (sagittal plane) to occupy more than 2/3 of the monitor screen, and endometrial thickness (ET) was measured to one decimal at the thickest part (double layer) [11]. Any echo-free space if present within the endometrial echo complex (EEC) was excluded. The ultrasonic examination was performed on 8–11 days of menstrual cycle [12]. In those with continuous bleeding, it was attempted to subsequently control bleeding with antifibrinolytic drugs subjecting them to sonographic evaluation after 8 days and not later than 11 days. Subjects, in whom bleeding was not controlled with antifibrinolytic drugs, were excluded from the study. Any finding on TVS outside the normal definition of EEC was assumed significant.

#### 2.2.2. Doppler Flow Study

After TVS examination, colour Doppler was activated and haemodynamic parameters were bilaterally analysed from the main ascendant branch of uterine arteries at the level of the cervical-corporal section of the uterus. The Doppler parameters measured were resistance index (RI = *V*_*S*_ − (*V*_*D*_/*V*_*S*_)) and pulsatility index (PI = *V*_*S*_ − (*V*_*D*_/*V*_*M*_)). *V*_*S*_ represents maximal systolic velocity, *V*_*D*_ represents end-diastolic velocity, and *V*_*M*_ represents mean average velocity during systole and diastole. Three identical waveforms of good quality were measured to determine the average value of pulsatility index (PI) and resistance index (RI) of both sides. A colour flow study of endometrial vessels was also performed to detect the number, size, and regularity of vessel branching. Three different vascular patterns were defined according to the Doppler flow mapping: (a) single vessel pattern is characteristic of the endometrial polyp. (b) Scattered vessel pattern is characteristic of endometrial hyperplasia. (c) Multiple vessel pattern is characteristic of endometrial carcinoma.

#### 2.2.3. Endometrial Sampling

All patients were subjected to endometrial sampling after the ultrasound examination. It was performed at different intervals with respect to the distinct pattern of menstrual bleeding. Endometrial sampling was performed (25–27 days) during the premenstrual period in patients with cyclic bleeding cases. In patients with atypical bleeding cases, the procedure was undertaken immediate postmenstrual. It was performed on the same day in patients with continuous bleeding. Patients were asked to empty their bladder. Under strict aseptic precaution, a pelvic examination was performed to know the size and position of the uterus. The posterior wall of the vagina was retracted by sims bivalved vaginal speculum, and the anterior lip of the cervix was caught by the vulsellum. The uterine sound was passed to know the position and length of the uterus. Endometrial material was aspirated by Carmen's cannula number 4 and sent for histopathological examination in 10% formalin. The histopathological samples were examined by a senior pathologist who determined the final diagnosis.

### 2.3. Statistical Analysis

Categorical variables were presented in number and percentage (%), and continuous variables were presented as mean ± SD and median. Normality of data was tested by Kolmogorov-Smirnov test. If the normality is rejected, then a nonparametric test was used. Quantitative variables were correlated using the independent *t-*test/Mann-Whitney test (when the data sets were not normally distributed) between the two groups. Qualitative variables were correlated using the chi-square test/Fisher's exact test. The receiver operating characteristic curve was used to find out the cut off point of endometrial thickness for predicting hyperplasia or malignancy. A diagnostic test was used to calculate sensitivity, specificity, NPV, PPV, positive likelihood ratio, negative likelihood ratio, and corresponding pretest and post-test probabilities. The correlation was calculated by Pearson's *R* test. A *P* value of <0.05 was considered statistically significant. The data were entered in an MS Excel spreadsheet, and analysis was performed using Statistical Package for Social Sciences (SPSS) version 21.0.

#### 2.3.1. Sample Size Calculation

The prevalence of AUB widely varies from 5% to 65% among women of reproductive age [13]. Assuming a prevalence of AUB of 50% with desired precision of 12.5%, level of significance of 5%, and power of 80%, the total sample size taken was calculated to be approximately 70.

## 3. Results and Observation

This observational study included 70 perimenopausal women with AUB who underwent TVS measurement of ET and Doppler study followed by endometrial sampling and HPE. Out of them, 51 (73%) women had a normal diagnosis at HPE while 19 (27%) women had neoplastic histology either benign or malignant. They are accordingly categorised into two groups: group I and group II. The mean ages of women in the group I and group II were 44.25 ± 2.69 and 47.16 ± 3.25, respectively (*P* < 0.001). Obese women (BMI ≥30) were having a 4.56-fold (95% CI 1.36–15.38) higher risk of having abnormal endometrium (*P*=0.01). Parity (*P*=0.69) and prevalence of diabetes (*P*=0.211) and hypertension (*P*=0.323) were comparable in both the groups (Table 1). Significant predictive factors of endometrial pathology as evident from univariate logistic regression were included for multivariate analysis. This revealed age >45 years old, and obesity and ET 10.5 independently increase the chances of developing endometrial pathology. Women with age >45 years old have been found to have an odds of 4.82 (1.02–22.83) and *P*=0.06 to develop abnormal endometrium in AUB (Table 2).

**Figure 1:**
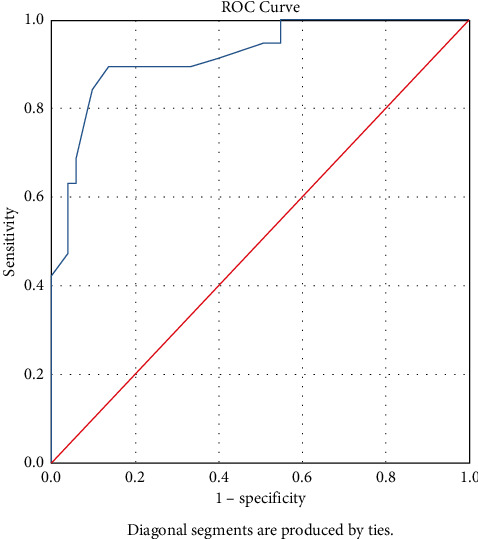
Receiver operating characteristic (ROC) curve for TVS-ET values obtained from all perimenopausal women with AUB. Area under the ROC curve (AUC) is 0.920 (95% confidence intervals 0.846 to 0.994, *P* < 0.001).

The ETs measured in group I and group II were 7.89 ± 2.62 mm and 14.07 ± 3.96 mm, respectively, with significant difference (*P* < 0.001). Our study revealed a TVS-ET of 10.5 mm has the highest sensitivity and specificity of 89.5% and 86.3%, respectively, and hence the highest Youden's index (Sensitivity + Specificity—1) as determined from receiver operating characteristic (ROC) curve (Figure 1). The area under curve (AUC) was 0.920 (95% confidence intervals 0.846 to 0.994, *P* < 0.0001). It has positive predictive value (PPV) of 70.68%, negative predictive value (NPV) of 95.68%, positive likelihood ratio (LR+) of 6.52 (with a pretest probability of 27% and post-test probability of 70.71%), and negative likelihood ratio (LR−) of 0.12 (with a pretest probability of 27% and post-test probability of 4.25%).

Out of 70 women, fibroid of any dimension was detected in 25 (35.71%) women at various locations (submucous, intramural, and subsubserosal) with an average ET of 8.95 mm. Endometrium was found thick (>12 mm) in 3 (4.28%) cases where one or more of any dimensions of the fibroid is more than 5 cm with the diagnosis of endometrial hyperplasia in all of them in HPE. The endometrial polyp was diagnosed in 6 (8.57%) women with an average ET of 9 mm. Other uterine pathologies diagnosed in TVS were adenomyosis in 14 (20%), myometrial cyst in 3 (4.28%), and chronic cervicitis in 3 (4.28%) women.

The mean resistance index (RI) and pulsatility index (PI) values of group I (0.89 ± 0.06, 2.11 ± 0.03) were higher than that of group II women (0.86 ± 0.06, 2.08 ± 0.08), respectively, but did not reach statistical significance. Colour flow study of endometrial vessels revealed positive findings in 33 patients, out of which 19 patients had normal endometrium on histology, 2 patients had an endometrial polyp, 8 patients had simple endometrial hyperplasia, 2 patients had complex endometrial hyperplasia, and 1 patient each had cystic glandular hyperplasia and endometrioid carcinoma. It has a sensitivity of 73.68%, specificity of 62.74%, PPV of 42.42%, NPV of 86.48%, LR+ of 1.98 (with a pretest probability of 27% and post-test probability of 42.26%), and LR− of 0.42 (with a pretest probability of 27% and post-test probability of 13.41%).

Histological examination in perimenopausal women revealed normal endometrium in 43 (61.42%), benign endometrial pathology (polyp, endometritis, and submucous fibroid) in 7 (10%), cystic glandular hyperplasia in 1 (1.42%), disordered proliferative endometrium in 1 (1.42%), simple hyperplasia without atypia in 13 (18.6%), simple hyperplasia with atypia in 2 (2.85%), complex hyperplasia in 2 (2.85%), and endometrioid adenocarcinoma in 1 (1.42%).

## 4. Discussion

Perimenopause is a period of transition due to a decline in ovarian function. There are systemic hormonal changes, which are evidenced by an early and late stage. The early stage is mostly marked by irregular cycles followed by a second stage of increasing longer periods of amenorrhoea of 60 days or more [2]. This hormonal milieu causes varied bleeding patterns, thus posing a difficult situation for clinicians to differentiate normal from pathological causes and, hence, the predicament for determining the endometrial cut off thickness in these women to diagnose endometrial pathology.

Histopathological examination of the endometrium is generally the principal mode of investigation to search for the cause of AUB, especially in women more than 45 years of age. However, this gold standard diagnostic modality may not be required in all women with AUB and should be offered to women at high risk for hyperplasia or malignancy [14]. The dilemma in individualising these women at risk, especially during perimenopause, motivates us to undertake the study. Our study reveals obese women with TVS-ET of 10.5 mm and above with complaints of AUB should undergo endometrial sampling. This critical value of ET had the sensitivity and specificity of 89.5% and 86.3%, respectively, to diagnose abnormal endometrium. We discovered that Doppler flow velocimetric study of endometrial and uterine vessels does not complement its diagnostic evaluation.

Age had been deciding criterion for a few academic societies for offering women with AUB for endometrial biopsy. The American College of Obstetricians and Gynaecologists and the National Institute for Health and Care Excellence advocated sampling of the endometrium as the initial mode of investigation in women above 45 years old [15, 16]. The Society of Obstetricians and Gynaecologists of Canada advocates that those women older than 40 years and having one or more risk factors such as BMI ≥30 mg/kg^2^ or nulliparity should also undergo endometrial biopsy [17]. There are, however, scarce studies to evaluate a limited cut off value of ET to diagnose endometrial pathology in perimenopausal women. In an observational cross-sectional study in 62 perimenopausal women more than 40 years of age, Mayuri et al. found sensitivity, specificity, PPV, and NPV of 90.9%, 87.5%, 80.0%, and 94.5%, respectively, to diagnose abnormal endometrium at a cut off endometrial thickness of ≥8 mm for perimenopausal women [18]. In another study comprising of 51 premenopausal patients with complaints of irregular bleeding, Smith et al. observed sensitivity of 67%, specificity 75%, positive predictive value of 14%, and negative predictive value of 97% assuming >8 mm endometrial thickness as cut off value [19]. In the third study in 177 women with peri- and postmenopausal bleeding who underwent vaginosonographic examination followed by HPE of the endometrium, Tongsong *T* et al. found an ET of 7 mm and less to reliably predict normal endometrium (100% sensitivity and 46% specificity) and hence advocated to offer the option of diagnostic curettage to women with thick endometrium (≥7 mm) [20]. However, a recent study in 240 premenopausal women with AUB by Luca et al. [21] observed the best endometrial thickness cut off value for the prediction of endometrial hyperplasia or endometrial cancer was >11 mm, which was almost similar to our study. Hence, the cut off ET values varied in different studies with failure to establish a diagnostic analogy, which probably is the reason that no definite value of ET had ever been determined in menstruating women with AUB. None of the critical values decided in the cited studies above had been performed with optimal standardisation of ultrasound schedule in regard to the menstrual cycle.

Numerous studies have been undertaken to evaluate the role of TVS Doppler in the detection of endometrial malignancy with conflicting results. Sawicki et al. found abnormal low impedance and high-velocity flow in the majority of the cases of endometrial cancer by transvaginal colour Doppler [10], while Mine et al. concluded the limited use of TVS Doppler due to moderate sensitivity and moderate-to-high specificity [9]. We discovered that both mean PI and RI values were low in neoplastic endometrial pathology with differences between the groups just being short of statistical significance. A study suggested the limit value for PI of 2.0 to predict endometrial cancer [22]. The average values in both the groups in our study were above 2.0, and the sensitivity of diagnosing endometrial lesions was low with Doppler. We speculated that our failure to detect any significant difference between the groups through colour Doppler could be due to the inclusion of all causes of AUB in the analysis like fibroid, adenomyosis, polyp, and endometritis having the potential to interfere with pelvic blood flow. Since only one case of adenocarcinoma was diagnosed and the rest being mostly hyperplastic endometrium, the subtle differences in blood flow indices could not draw statistical significance. However, power Doppler application, especially endometrial vascularisation index (VI), may be useful for discrimination between normal and malignant endometrium in premenopausal women with abnormal uterine bleeding evident from studies [23, 24], which we could not undertake due to technical limitations.

The significant difference in the mean age of both the groups in our study reveals that higher age is a risk factor for endometrial pathological lesions. This mean age incidence in the groups is similar to another study undertaken in Indian women [18]. Many studies have associated increasing age and parity with a higher incidence of AUB [25, 26]. However, we found parity comparable in both groups. We also discovered obesity as a risk factor for the development of abnormal endometrium. This discovery in Indian women is consistent with global data reporting 40%–45% of cases of endometrial carcinoma in Europe [27] and 57% in the United States [28] to be associated with obesity. Obesity leads to complex variance in the levels of hormones and different factors of metabolism through the generation of oestrogen from adipose tissue by aromatisation, thereby substantially increasing the risk of endometrial hyperplasia and endometrial carcinoma [29]. In perimenopausal women, in the absence of ovulation, the exposure of endometrium to oestrogen can further augment this risk in obese women.

The strengths of our study are subject preference and study methodology. Very few studies have been undertaken in perimenopausal women to determine ET, and our study will definitely guide clinicians to classify pathological endometrium to subject them to invasive sampling. Since the variation in ET is evident throughout the menstrual cycle of a woman, we tried to ensure uniformity in the schedule of ultrasound examination by measuring the endometrial thickness on 8–11 days of the menstrual cycle. This standardisation in ultrasound timing in women with cyclical and in atypical bleeding cases will avoid measuring false thick endometrium at a later stage. Since we included women with AUB for all causes, this study depicts the precise presentation in the true clinical setting. The limitations of the study include our failure to undertake the hysteroscopic examination of the uterine cavity. The drawbacks of blind endometrial biopsy along with the advantage of direct visual inspection of endometrial pathology cannot be overlooked.

Hence, we conclude women in perimenopause and AUB should be offered to undergo endometrial sampling for histopathological examination if TVS-ET is 10.5 mm and above when performed in the first 10 days of the menstrual cycle. Doppler complements its diagnostic accuracy; however, a negative finding should not deter further investigation. The coexisting risk factors, especially higher age (>45 years) and obesity (BMI>30), significantly escalate the chances of developing endometrial pathology.

## Figures and Tables

**Table 1:** Characteristics of patients.

	Group I (*n* = 51)	Group II (*n* = 19)	*P* value
Age (mean ± SD)	44.25 ± 2.69	47.16 ± 3.25	0.000^a^
Parity (mean ± SD)	2.89 ± 1.48	2.75 ± 1.35	0.690^a^
HTN present (*n*, %)	6 (11.76%)	4 (21.05%)	0.323^b^
Obesity (*n*, %)	7 (13.72%)	8 (42.10%)	0.010^b^
Diabetes (*n*, %)	5 (9.80%)	4 (21.05%)	0.211^b^
BMI (mean ± SD)	22.56 ± 4.09	23.68 ± 4.65	0.330^a^
Mean TVS-ET	7.89 ± 2.62	14.07 ± 3.96	0.000^a^
Mean RI values	0.89 ± 0.06	0.86 ± 0.06	0.084^a^
Mean PI values	2.11 ± 0.03	2.08 ± 0.09	0.065^a^

^a^Independent *t*-test and ^b^chi-square test.

**Table 2:** Univariate and multivariate logistic regression analyses of factors for prediction of endometrial pathology.

	Group I (*n* = 51)	Group II (*n* = 19)	*P* value (univariate)	OR	*P* value (multivariate)	AOR
Age >45	21 (41.17%)	14 (73.68%)	0.016	3.95 (1.25–10.12)	0.06	4.82 (1.02–22.83)
Obesity (BMI ≥30)	7 (13.72%)	8 (42.10%)	0.010	4.56 (1.36–15.38)	0.03	4.23 (0.82–21.60)
HTN^*∗*^	6 (11.76%)	4 (21.05%)	0.323	0.500 (0.12–2.01)	—	—
Diabetes^*∗*^	5 (9.80%)	4 (21.05%)	0.211	2.45 (0.58–10.30)	—	—
ET >10.5	14 (27.45%)	15 (78.94%)	0.000	26.78 (5.56–44.48)	0.004	22.60 (1.91–47.93)

^*∗*^Multivariate analysis is performed for only significant values in univariate analysis.

## Data Availability

The data used to support the findings of this study are available from the corresponding author upon request.
